# The Emerging Role of Mitochondrial Dysfunction in the Pathogenesis of Idiopathic Inflammatory Myopathies

**DOI:** 10.5041/RMMJ.10493

**Published:** 2023-04-30

**Authors:** Jorge Armando Gonzalez-Chapa, Marina Barguil Macêdo, Christian Lood

**Affiliations:** Division of Rheumatology, University of Washington, Seattle, WA, USA

**Keywords:** Dermatomyositis, inclusion body myositis, mitochondria, muscular diseases

## Abstract

Increasing evidence points towards mitochondria as crucial players in the initiation and progression of auto-immune and degenerative disorders, to which impaired cell metabolism is but a facet of the subjacent etiopathogenesis. This review aims to introduce the reader to essential concepts of mitochondrial abnormalities in idiopathic inflammatory myopathy (IIM), underscoring inclusion-body myositis and dermatomyositis. Far surpassing the initial simplistic view of being responsible for energy generation, mitochondria have gathered attention regarding their role in inflammatory processes, being able to fuel autoimmunity, as shown by the presence of anti-mitochondrial antibodies (AMAs) in up to 10% of IIM patients. As cellular respiration takes place, mitochondrial metabolites might help to shape the pro-inflammatory milieu in affected muscle, beyond generating reactive oxygen species, which are well-recognized inducers of damage-associated molecular patterns. A series of mitochondrial components might facilitate the sterile activation of pro-inflammatory cells and the production of several cytokines responsible for enhancing auto-immune responses. Marked variation in the mitochondrial genome has also been reported in IIM patients. As such, we summarize key historical and recent advances linking aberrations and instabilities of mitochondrial DNA to impaired muscle function. Besides discussing mitochondrial dysfunction as an essential part of IIM development, we also highlight possible associations between presence of AMAs and a particular phenotype of IIM, with its own characteristic clinical and radiological pattern. Finally, we present promising treatment approaches targeting mitochondria, while briefly discussing experimental models for gaining deeper insight into the disease process, and ultimately leading to novel drug development.

## INTRODUCTION

Mitochondrial abnormalities as a source of muscular disease have been described over six decades ago, when it was first shown that dysregulation of cellular metabolism by impairment of oxidative phosphorylation was the central mechanism of muscle hypotony in a unique group of diseases that would be later labeled as “mitochondrial myopathies.”^1^ Mitochondrial myopathies are under the category of inborn errors of metabolism diseases, also known as congenital metabolic disorders, being peculiar regarding its inheritance pattern, as a solely maternally transmitted disease, as mitochondrial deoxyribonucleic acid (mtDNA) is inherited exclusively from the mother.^2^

As the preponderant role of mitochondria in the cell function and dysfunction of skeletal muscle has been discovered, it became evident that, beyond being at the core of the pathophysiology of mitochondrial myopathies, mitochondrial disturbances are a shared feature of myopathies in general, occurring either primarily, as in the case of mitochondrial myopathies, or secondarily in the disease course, *id est*, representing either the cause or the consequence of myopathic changes.^3^,^4^

Mitochondrial dysfunction may either be primary (genetic) or secondary (acquired), resulting in an insufficient number of mitochondria per cell, and thus restricting the cellular metabolic capacity. By negatively impacting cell homeostasis, mitochondrial dysfunction may trigger the development and establishment of autoimmune and degenerative disorders. Several treatments are being tested to reverse this dysfunction, including not only inflammation-reducing therapies, but also those targeting metabolism restoration, such as infusion of healthy mitochondria into tissue, currently in clinical trial.

That mitochondria perform a leading role in muscle health and disease is not unexpected, as the muscle is the human tissue that harbors the greatest number of mitochondria per cell, whose ratio may range to up to tens of thousands in some instances, representing up to 5% of the muscle cell volume.^5^ Those massive numbers are needed to support muscle bioenergetics: being a highly metabolic active tissue, skeletal muscle is responsible for around 30% of resting energy expenditure in humans.^6^

Advancements in the understanding of mitochondrial dysregulation have been fostered by the standardization of the technique of staining muscle biopsy samples for the mitochondrial component cytochrome c oxidase (COX).^7^ The COX-negative fibers turned out to be a robust marker of mtDNA dysfunction, being consistently but not exclusively found in inborn errors of metabolism diseases, since inclusion body myositis (IBM) samples also characteristically present an increased number of COX-negative fibers as compared to age-matched controls.^8^ The realization of this contributed to the gathering interest about how the collapse of mitochondrial homeostasis may be the very essence of myopathy development.^9^

Moving forward, this interest expanded to the field of myositis, that is to say, of myopathy with inflammatory features. As mitochondria became recognized as more than simply the “cell’s powerhouse,” also playing a chief role in oxidative stress-induced inflammation and cell death, their impact on autoimmune disorders came into the limelight.^10^ Literature on this topic has accrued steadily over the past decade, but still, there is much to be explored. Herein, we propose to review the most recent and emerging concepts surrounding the intricate relationship between mitochondria dysfunction and idiopathic inflammatory myopathies (IIM).

## MITOCHONDRIAL DNA GENETICS

### Mitochondria and Regulation of Its DNA

Human mtDNA is composed of a double-stranded circular molecule organized into a heavy guanine-rich strand and a light cytosine-rich strand.^11^,^12^ The small mitochondrial genome is built of approximately 15,569 base pairs and contains 37 genes that are responsible for encoding 13 mitochondrial proteins, 22 transfer ribonucleic acids (tRNAs), and 2 ribosomal RNAs (rRNAs).^2^ Mitochondrial DNA contains about 95% of coding regions.^12^ In contrast, nuclear DNA has approximately 2% of coding regions.^13^ The main non-coding section of mtDNA has been named the displacement loop and is composed of 1,123 base pairs. This is approximately 7% of the mitochondrial genome and represents the major control site of mtDNA replication and expression.^11^,^12^

Although mitochondria have their own mtDNA, they are not self-sufficient. Only 13 of about 1,500 mitochondrial proteins are encoded by the mtDNA.^14^ These proteins are part of complex I (ND1–ND6 and ND4L), complex III (CYTB), complex IV (COX1–COX3), and complex V (ATP6 and ATP8). All subunits involved in complex II, as well as the remaining ~1,500 proteins, are encoded by nuclear DNA, and are later translocated through the mitochondrial membrane.^15^,^16^ This is particularly relevant as some cytosolic proteins, including amyloid precursor protein,^17^ may mislocalize to mitochondria, accumulate in important mitochondrial membrane proteins such as TOM and TIM23, and thus impair the import of molecules indispensable for mitochondrial function.^18^,^19^ The subsequent proteolytic cleavage of amyloid precursor protein may result in the formation of amyloid-β peptides, which can also interfere with this process, and even accumulate in the mitochondrial matrix impairing normal mitochondrial function.^20^,^21^ This is extremely relevant in diseases associated with amyloid-β peptide aggregation such as in IBM,^22^ amyotrophic lateral sclerasis,^23^ and Alzheimer’s disease.^24^

Inside the mitochondrial matrix, the mtDNA is organized in the form of nucleoids, which have a uniform size and usually contain a single copy of mtDNA compacted by mitochondrial transcription factor A.^25^–^27^ In healthy individuals, this genetic information is mainly present in a homoplasmic way. This means that the mitochondrial genomic information is mainly associated with one allele. Some mutations might be present without having a direct implication on human health. However, if these mutations reach a certain threshold, the balance will be lost, and a high heteroplasmy level will be reached.^28^,^29^

It is important to note that the mitochondrial division cycle is independent of the cell cycle. Thus, different cells can have different amounts of mtDNA, causing a mosaic pattern in tissue.^30^ This is of particular interest in patients with certain myopathies, such as IBM in which the accumulation of abnormal mitochondria with a high mtDNA copy number will create the so-called “ragged muscle fiber” in muscle biopsies.^31^ This pattern helps to differentiate it from dermatomyositis (DM), in which the frequency of these fibers is low.^32^

### Mitochondrial DNA Deletions

Several factors make mtDNA susceptible to suffering mutations, in particular the lack of protective histones, an inefficient DNA repair system, and close proximity to reactive oxygen species (ROS) generation. All of these together result in mtDNA having an almost 10 times higher basal mutation rate than nuclear DNA.^32^–^35^

Mutations in mtDNA can be primarily divided into point mutations, also called single nucleotide polymorphisms, and deletions.^30^ Deletions in mtDNA involve the loss of certain segments of the mitochondrial genome. They are frequently observed in IBM, with studies reporting it in up to 60% of affected individuals.^36^ Some of the most common mtDNA deletions in IBM have shown an approximately 10% level of heteroplasmy when compared to healthy individuals. These deletions are mainly associated with three specific regions: m.534–4429, m.6330–13993, and m.8636–16072.^37^,^38^

Most of these deletions occur in a specific part of the mitochondrial genome named “common deletion,” or “mtDNA^4977^,” which eliminates nucleotides between 8470 and 13447 of the mitochondrial genome.^39^,^40^ Consequently, the destruction of about one-third of the whole mtDNA might be affected by this mutation. This particular mtDNA deletion is not selective for, but highly enriched in, IBM patients, being also seen in other conditions, including Alzheimer’s disease, malignancies, ultraviolet light-injured skin, autosomal dominant progressive external ophthalmoplegia, as well as in normal aging.^39^,^41^–^43^ Contrary to IBM, very low numbers of large-scale mtDNA deletions have been reported in DM: less than 0.05% of DM muscle biopsies present this mutation, not differing from control subjects regarding this aspect.^44^

Although mtDNA deletions occur more frequently in IBM patients, they are not unique deletions, but rather mtDNA deletions associated with normal aging and sarcopenia.^45^–^47^ Thus, it is worthwhile considering an accelerated aging process being involved in IBM pathogenesis, promoting mtDNA deletions and subsequent dysfunction.

The physiological importance of mtDNA^4977^ is the presence of essential mitochondrial genes on the compromised segment, including five tRNA genes (tRNA^Gly^, tRNA^Arg^, tRNA^His^, tRNA^Ser^, and tRNA^Leu^); seven genes responsible for encoding subunits ND3, ND4, NDL, and partially ND5, which all are part of complex I; COX II, which is a complex IV subunit; and ATP6 and ATP8, which belong to complex V. Any large deletion in this region might lead to serious consequences in the mitochondrial oxidative phosphorylation function.^39^

These deletions have been reported to be particularly common in the peripheral regions of the IBM muscle fibers. Deletions might be present in contiguous segments of the same muscle fiber, so mutant mtDNA might focally expand causing a specific segment COX deficiency.^48^,^49^ Similar to IBM, mtDNA copy number has been reported to be significantly reduced also in DM muscles, particularly in the perifascicular regions. Even more, these reports provide evidence that respiratory chain dysfunction in DM is associated with mtDNA depletions.^44^,^50^,^51^ Such COX-deficient fibers have also been associated with DM, lacking complex I and IV, while having preserved complex II activity.^44^

### Mitochondrial Gene Variants

Several mtDNA single nucleotide polymorphisms, mainly located in the displacement loop, have been found to be enriched in cancer,^52^ rheumatoid arthritis,^53^ and systemic lupus erythematosus.^54^ Though IBM patients primarily have mtDNA deletions, as discussed above, several mtDNA gene variants have also been implicated, including m.1438A>G, m.1382A>C, and m3849G>A.^38^,^55^ Among these, m.1438 might be of particular relevance since it has been related to other diseases, such as type 2 diabetes^56^ and schizophrenia.^57^ Though this gene variant is upstream of ND1, the potential impact on mitochondrial function is not known, and protein expression levels of ND1 remain similar.^55^

In contrast to IBM, several gene variants have been described in DM, especially in the mitochondrial displacement loop. Two main alleles in this location may be representing an important pathological association in these patients, 16304T/C and 16519T/C, with the latter being associated with positive antinuclear antibody status, as well as interleukin-2 production.^58^ Thus, this particular gene variant, and/or haplogroup, may be implicated in loss of self-tolerance, promoting autoantibody generation in those individuals.

Finally, since the mitochondria are under dual control, relying on both nuclear and mitochondrial DNA, mutations in either of them may directly affect mitochondrial function which can subsequently lead to further mtDNA mutations.^59^,^60^ Therefore, several studies have attempted to find a relationship between nuclear gene variants involved in mitochondrial maintenance and stability. Of particular interest is *POLG1*, which is responsible for the synthesis and repair of the mitochondrial genome.^30^,^61^ Nonetheless, so far, no nuclear DNA gene variants have been associated with enhanced mtDNA mutation rate in patients with myopathies.^38^,^50^,^62^,^63^

In summary, mtDNA mutations are present in both IBM and DM, with IBM being characterized by mtDNA deletions, and DM more frequently having mtDNA gene variants. Though the exact consequences of the mtDNA gene variants are unknown, it is anticipated that they might result in aberrant mitochondrial function, reducing oxidative phosphorylation and/or amplifying ROS generation, causing local energy deprivation, inflammation, and damage in muscle. Further studies are needed to characterize the functional outcome of the mtDNA gene variants and deletions, as well as their role in IBM and DM pathogenesis.

## METABOLISM

Muscles are responsible for about 20% of the resting metabolic rate, and can quickly increase their metabolic rate 100-fold during intense exercise.^64^,^65^ Consequently, due to its high metabolic need, muscle tissue is very sensitive to metabolic changes. Thus, mitochondrial impairment (either through environmental changes, or mtDNA mutation and/or heteroplasmy) could lead to decreased adenosine triphosphate (ATP) production, increased ROS generation, and subsequently muscle weakness, fatigue, and other myopathic symptoms.^22^,^66^,^67^ However, very little is known about the muscle metabolic activity in DM or IBM.

Recent work has highlighted primary respiratory chain deficiencies in IBM patients,^68^ with elevated levels of cystathionine, dimethylglycine, taurine-conjugated taurochenodeoxycholic acid, and citrulline in peripheral blood. Furthermore, kynurenine and its hydroxylated form, 3-hydroxy-DL-kynurenine, which represents the alternative nicotinamide adenine dinucleotide synthesis pathway, were found to be upregulated, with a reduction in niacinamide levels. In addition, a low global creatine pool, represented by an increased creatine/creatinine ratio, was also found. Finally, IBM patients were found to have increased levels of nucleotide synthesis precursors such as adenosine, deoxycytidine, cytidine, and cytosine, as well as carbohydrate derivates such as sucrose and myoinositol. Thus, at least in peripheral blood, IBM patients have marked metabolic abnormalities. Limited insight from skeletal muscle indicates normal oxidative metabolism rate in IBM.^69^ Future studies are warranted to determine the metabolic activity in tissue. If validated, therapies targeting metabolic activity, such as nicotinamide adenine dinucleotide boosters, or rapamycin, may be considered, given their beneficial effect in an experimental myopathy model.^70^–^72^

In DM, patients have reduced ATP production and proton rate efflux from muscle fibers, as well as twice as long recovery half-time of phosphocreatine and adenosine diphosphate after exercise.^73^ Some of these findings may relate to intrinsic mitochondrial abnormalities, e.g. mtDNA gene variants, described above. Mitochondrial gene variants leading to reduced ATP production are associated with decreased intracellular acidosis and a normal to increased rate of H^+^ efflux from the cell during exercise.^74^,^75^

It is important to also recognize the essential role of the vasculature in providing oxygen to tissue in DM. Patients with DM have reduced capillary density, with focal capillary depletion, related to complement membrane attack complex formation,^76^,^77^ which may contribute to causing local hypoxia and further inflammation.^78^ Reduced H^+^ efflux rate and an affected oxidative phosphorylation have been shown in ischemic conditions^79^ and in peripheral vascular disease.^80^ In contrast to DM, IBM patients have normal capillary density and no involvement of the complement system.^77^

In summary, there are emerging data suggesting aberrant metabolism in both IBM and DM, either in blood or in muscle. Future studies are needed to understand what is the subjacent cause, as well as what are the functional consequences.

## MITOCHONDRIAL-MEDIATED INFLAMMATION

Mitochondria, given their prokaryotic origin, contain several danger-associated molecular patterns, including their highly methylated DNA, cardiolipin, N-formyl methionine peptides, among others, able to trigger local inflammation and immune cell infiltration.^81^ However, the exact roles of these danger-associated molecular patterns in myopathies are not fully elucidated.

We, and others, have recently highlighted an important role for N-formylated methionine peptides in several rheumatic diseases, including scleroderma, rheumatoid arthritis, and vasculitis,^82^–^84^ with elevated levels of N-formyl methionine peptides in peripheral blood promoting neutrophil activation through FPR1-mediated pathways. However, whether mitochondrial-derived N-formyl methionine would be released by damaged/dying muscle tissue in myopathies, and cause local and/or systemic inflammation, has not yet been studied.

Cardiolipin, a mitochondrial-derived phospholipid on the inner mitochondrial membrane, can, upon oxidative damage, translocate to the outer mitochondrial membrane and trigger NOD-like receptor family pyrin domain containing 3 (NLRP3)-mediated inflammasome activation.^85^ Of note, local inflammasome activation, with interleukin-1β and interleukin-18 production, is a common feature in the muscle of DM patients.^86^ However, whether this is driven through mitochondrial ROS and cardiolipin is not known.

Release of mtDNA can take place either intracellularly, as upon mitochondrial damage caused by transactivation response DNA-binding protein 43 (TDP-43) aggregates,^87^ or extracellularly, as may occur upon cell death.^88^–^90^ Once released, mtDNA can be recognized by DNA sensors in endosomes,^91^ and/or in cytosol,^88^ leading to induction of pro-inflammatory cytokines, primarily type I interferons.^81^ Even though type I interferons are prominently up-regulated in DM,^92^ and toll-like receptor 4 and toll-like receptor 9 have been shown to promote inflammation in myopathies,^93^,^94^ the part that mitochondria take in these processes is not understood. Given that type I interferons promote vascular damage,^66^,^76^,^95^ and reduce mitochondrial respiration in myotubules,^95^ both key processes in DM, future studies are needed to better characterize the mechanism of type I interferon induction in DM, both the receptor(s) and agonist(s), to enable the development of novel therapies.

Also, it is noteworthy that, despite the reasonable assumption that mutated mtDNA reported in IIM may make the myocytes more sensitive to apoptosis,^62^ this type of cell death is not a common finding in muscle cross-sections of either IBM or polymyositis (PM).^96^ This is in stark contrast to the positivity, observed on immunohistochemistry, of Fas and Fas ligand in affected myofibers.^96^,^97^ This discrepancy is likely accounted for by the concomitant high expression of the B-cell lymphoma 2 protein (Bcl-2), which inhibits apoptosis.^96^ The reason for the up-regulation of this mitochondrial protein, though, is not clear. And while one may instinctively regard such a mechanism as protective to the myocyte, in truth it may actually perpetuate muscle injury, since it also occurs in infiltrating T cells: they, too, present the same apoptosis-resistant feature, rendering them resistant to treatment with immunosuppressants.^98^

Intriguingly, COX-deficient fibers (e.g. those having mitochondrial abnormalities) are associated with up-regulation of major histocompatibility complex class I and infiltration of T cells into muscle tissue in DM and IBM, respectively.^44^,^99^,^100^ Currently, causality is not proven, and whether COX deficiency precedes inflammation or whether it is a consequence of the inflammation remains to be clarified. Inflammatory cytokines, including chemokine (C-X-C motif) ligand (CXCL)-8, interleukin-6, tumor necrosis factor-α, interleukin-1β, and monocyte chemoattractant protein-1, have been implicated in mitochondrial damage and mitochondria–muscle cross-talk.^101^,^102^ However, more studies are needed to elucidate their role in the pathogenesis of DM and IBM.

Despite approximately 90% of the cellular ROS production being generated in mitochondria,^103^ endogenous sources such as peroxisomes, endoplasmic reticulum, and even some metabolic processes can contribute to ROS formation.^104^ Intriguingly, ROS can also be produced by exogenous stimuli, including alcohol, tobacco smoke, paracetamol, doxorubicin, metronidazole, high temperatures, and ultraviolet light.^105^

During the last few years, ROS has been mainly considered as a purely toxic species contributing to different diseases, such as myopathies, and other conditions, including aging. However, the direct intracellular and mitochondrial effect of ROS depends on many variables. Among these are the site of ROS production, its persistence, and the cell’s antioxidant status. In light of these variables, ROS signaling pathways can be controlled by a dynamic scenario that will cause a physiological or pathological response in the muscle fibers. This dynamic variability can be exemplified by brief, low ROS levels encouraging mitochondriogenesis, mitochondrial function, muscle adaptation and differentiation, and overall cell survival. In contrast, a persistent, high ROS production causes mitochondrial dysfunction and mutations, apoptosis, autophagia, muscle atrophy, and inhibition of muscle fiber differentiation, repair, and regeneration.^106^

Although increased oxidative damage participates in the pathogenesis of mitochondrial myopathies, data indicate that ROS might be just one of several components. This was demonstrated by the attenuation of mitochondria superoxide production in skeletal muscle fibers by SS31 tetrapeptide, which reduced mitochondrial ROS and prevented oxidative damage. Regardless of how this antioxidant treatment caused certain benefits, it failed to redeem the muscle fibers from a sarcopenic phenotype associated with atrophy and mass loss, suggesting that the redox environment might not be the key regulator during a degenerative process.^107^ Oxidative stress has been considered to be one of several components in the progression of degenerative diseases and mitochondria-related muscle pathologies, such as IBM and DM. However, whether the myopathic changes in these diseases are a cause or a consequence of ROS alterations is still under discussion, and needs further investigation.

Another class of signaling molecules with cytokine-like properties might be produced and released directly from the mitochondria, namely mitokines. Depending on the context, they might have pro- or anti-inflammatory effects, and play important roles in a wide range of cellular processes, such as cell inflammation, apoptosis, metabolism, and cell survival.^108^ Some of the best-studied mitokines are humanin, fibroblast growth factor 21, and growth differentiation factor 15.^108^ Serum levels of fibroblast growth factor 21 have been reported to be normal or slightly elevated in IBM patients.^68^,^109^ Similarly, serum levels of growth differentiation factor 15 are increased in both IBM and DM patients.^55^,^110^,^111^ Growth differentiation factor 15 has been proposed as a useful marker for oxidative phosphorylation deficiencies, since it has been shown to be increased in patients with mitochondrial defects.^112^ Understanding the role of mitokines in health and disease is still an active research area, and much is still unknown regarding the complex mechanisms of interactions between mitokines and cells.

## AUTOANTIBODIES

The presence of autoantibodies is frequent in, though not a requirement to, the diagnosis of IIM. In fact, multiple cohorts have shown that myositis-specific antibodies (MSA) or myositis-associated antibodies are positive in around 60% of IIM patients.^113^,^114^ While MSA are helpful for both establishing and classifying IIM diagnosis, myositis-associated antibodies, in turn, are common to several other autoimmune disorders, being thus supportive, but non-specific, of IIM diagnosis.^115^ Remarkably, MSA have the particularity of being almost mutually exclusive, the co-existence of two or more in the same patient being exceedingly rare.^116^ Among MSA, anti-Jo1, an antibody directed against anti-histidyl-tRNA synthetase, is the one best described and for which a standardized reliable test is more readily accessible. Reflecting its high predictive positive value, the presence of anti-Jo1 in serum confers the highest individual score point on the European Alliance of Associations for Rheumatology (EULAR)/American College of Rheumatology (ACR) 2017 Classification Criteria for IIM.^117^

Anti-mitochondrial antibodies (AMAs) belong to the category of myositis-associated antibodies. Their presence is uncommon in IIM patients, perhaps because they are, in most instances, not pro-actively searched for, as they lack specificity for IIM diagnosis. Another hindrance to more widespread testing is that the recommended validated technique is indirect immunofluorescence, which requires specialized laboratories to be performed.^118^ Of note, in essence, the terminology “AMA” actually refers not to one but to several autoantibodies against different lipoic acid complexes inside the inner mitochondrial membrane, but the one routinely tested for is the E2 subunit of the 2-oxo acid dehydrogenase complex, also referred as AMA-M2.^119^

In a Japanese cohort in which all IIM patients were systematically screened for AMA, 11.3% were AMA-positive.^120^ Maeda et al. suggested that the presence of AMA may be correlated with increased frequency of cardiac involvement, manifesting as arrhythmia or decreased ejection fraction. The most striking feature of the AMA-positive patients, though, was that, in half of them, there was no report of muscle weakness—that is to say, they presented with clinically amyopathic disease.^120^

That AMA-positive IIM may have its own distinctive phenotype, consisting of less prominent striate muscle involvement, as opposed to more marked (and sometimes limited to) cardiac muscle involvement, is a hypothesis that deserves consideration. As evidence of that, there is a subsequent case report of a Japanese patient that manifested solely with recurrent atrial flutter, and who later received the diagnosis of IIM based on elevated creatine kinase levels and a characteristic muscle biopsy.^121^ Yet another case report describes a Japanese patient with AMA-positive isolated cardiomyopathy, that upon imaging study resembled cardiac sarcoidosis, which prompted the execution of a cardiac muscle biopsy which, surprisingly, evidenced IIM.^122^ Those findings, though interesting, need further replication, preferably in an ethnic-diverse cohort, as most of the available literature comes from East Asia.

Suggestively, nonetheless, an American case series of seven patients (two Afro-Americans and five Caucasians), indeed pointed out a possible association between AMA and heart involvement, with cardiac symptoms predating muscle symptoms in the majority of patients.^123^ Later on, the same research group published a more extensive descriptive study of the Johns Hopkins cohort, comprising almost a thousand individuals with IIM, of which 5% turned out to be AMA-positive. In that study, they confirmed that cardiac involvement was enriched in adult AMA-positive patients, but not in children who were AMA-positive. On the other hand, dysphagia was noticed as significantly more frequent both in AMA-positive adults and children.^124^ The authors stated, as a conclusion, that detection of AMA should instigate physicians to monitor for cardiac and gastroesophageal dysfunction.

The reverse should also be true: in a Danish case report of an AMA-positive IIM patient that evolved with rapidly progressive heart failure, and eventually succumbed to it, the authors urged physicians to order AMA testing in patients who manifest with unexplained heart dysfunction, even in the absence of striate muscle involvement. How the proper diagnosis may impact management, though, is not clear to date, as they acknowledged that cardiac and striate muscle response to immunosuppression may differ, the latter being more amenable to stabilize or improve under pharmacological treatment than the former.^125^ A requirement for surgical intervention and device implantation (such as a pacemaker or an implantable cardioverter defibrillator) for controlling cardiac symptoms is not exceptional, highlighting the value of a multidisciplinary team caring for the patient.^126^ Indeed, patients are best managed when being followed jointly by rheumatology, neurology, and cardiology teams.

As myositis-associated antibodies, AMAs may assist in the diagnosis of other concurrent autoimmune disorders, most often primary biliary cholangitis, as they are actually part of the diagnostic criteria for this disease.^127^ Historically, AMA presence has been deemed almost a synonym of primary biliary cholangitis, but only one-quarter to one-third of AMA-positive IIM patients also have primary biliary cholangitis.^120^ This notwithstanding, regular monitoring of alkaline phosphatase may be advisable, as it may uncover subclinical primary biliary cholangitis.^128^ Other autoimmune disorders that have been described in association to AMA-positive IIM are thyroidopathies (both Graves and Hashimoto), systemic sclerosis, Sjogren disease, and myasthenia gravis.^129^–^131^

Not only in regard to clinical phenotype may AMA-positive IIM have its own defining hallmarks. A case series of six Japanese patients demonstrated a peculiar high-intensity signal on the short-tau inversion recovery sequence of muscle magnetic resonance imaging on the adductor magnus, which the authors named the “cuneiform sign.”^132^ The authors emphasized the fact that this is an unusual pattern for IIM, as DM and PM habitually present with edema on short-tau inversion recovery sequence that is most prominent in anterior muscles, such as vastus lateralis and intermedius.^131^ However, they did not include a control group, so those results may not be generalizable.

**Figure 1:**
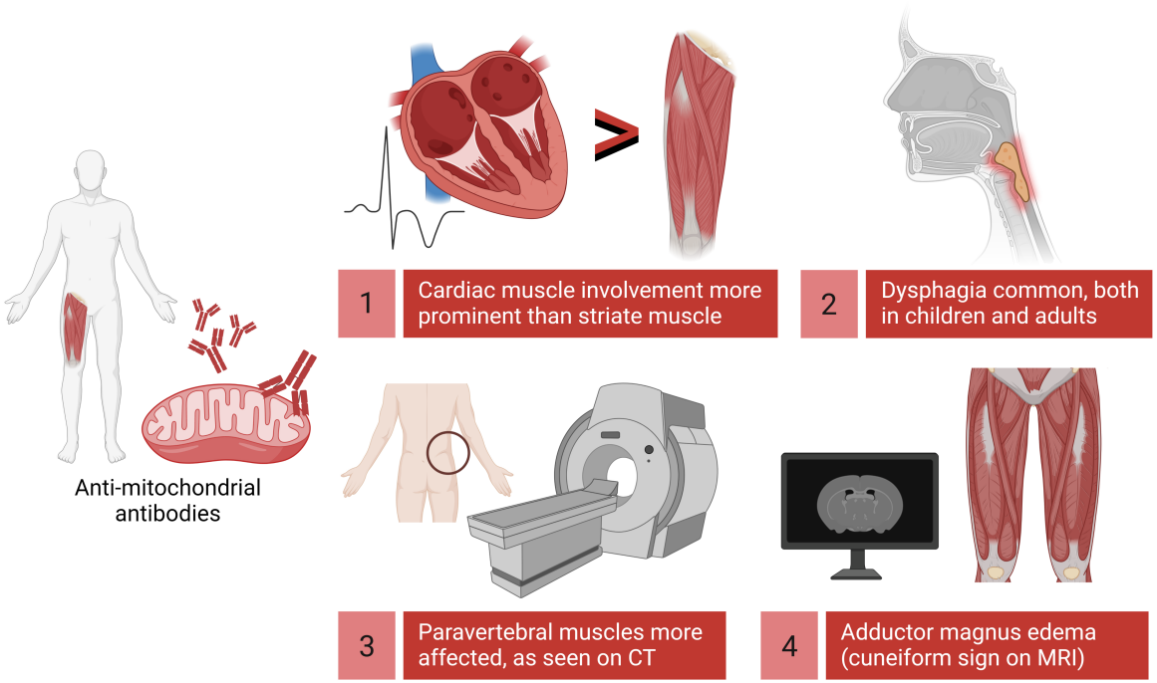
Clinical and Radiological Phenotype of Anti-mitochondrial Antibody-positive Idiopathic Inflammatory Myopathies. CT, computed tomography; MRI, magnetic resonance imaging. Created with Biorender.com.

A Japanese case-control study of eight AMA-positive IIM individuals, in contrast, highlighted the relative sparing of lower limb muscles on imaging, with atrophy being more intense in paravertebral muscles.^133^ The imaging method adopted in that study, however, was computed tomography, which is recognized as being more useful for demonstrating chronic than acute changes in muscle, and as such may not faithfully mirror the results seen on magnetic resonance imaging (Figure 1).

Concerning prognosis, evidence is conflicting. While there are studies showcasing the high morbidity and mortality attributed to fulminant cardiac involvement,^134^ there are others that stress that AMA-positive IIM is a steroid-sensitive disease, resulting in favorable outcomes when aggressive immunosuppression is promptly established.^135^

As a last remark, it should be noted that even though anti-cardiolipin antibodies are usually not tested when ordering AMA laboratory testing, they actually are, per definition, a subtype of AMA.^136^ Cardiolipin is one of the main components of the inner mitochondrial membrane, constituting around one-fifth of its lipid composition.^137^ Testing for anticardiolipin antibodies is not usually performed in the context of IIM, with only a few case reports describing the co-occurrence of anti-cardiolipin antibodies with other MSA/myositis-associated antibodies. Testing was driven by the appearance of deep vein thrombosis, which motivated the investigation of pro-thrombotic factors that could have led to this presentation, a rather atypical one for IIM.^138^,^139^

## TREATMENT

A treatment approach focusing on AMAs has not been thus far identified, even more so because, up until now, AMAs are considered more like an epiphenomenon than an actual pathogenic antibody influencing disease severity, and consequently they are viewed more as a diagnostic biomarker than a treatment target.^140^ As a disease group, IIMs tend to respond reasonably well to immunosuppressants, albeit relapses are quite common when drugs are tapered.^141^ Whether and how the immunosuppressants in current practice interact with AMAs remains, however, to be elucidated. Moreover, how AMA titers vary according to therapy remains a largely unexplored field, with the only evidence available coming from primary biliary cholangitis: an Asian cohort demonstrated that a decrease of AMA titers was noticed in patients responding to ursodeoxycholic acid therapy, but not in non-responders.^142^

In contrast, despite being categorized under the umbrella term of IIM, IBM follows a different disease course, showing poor or no response to immunosuppressants.^143^ Of interest, IBM typically shows on histopathology an extensive disturbance of mitochondria structure, in which impaired mitophagy seems to be involved, and as such may hold promise for the development of a drug that could enhance clearance of damaged mitochondria.^144^ In face of that, it is no surprise that the few published data on how treatment may reverse pathological mitochondrial changes come from IBM models. Further, as treatment for IBM is largely non-pharmacologic, concentrating on physical therapy and rehabilitation, evidence emerges mainly on the effect of exercise on mitochondrial dysfunction.^110^ Animal models have revealed that exercise is able to lessen the release of cytochrome c from mitochondria to the myocyte’s cytoplasm, reduce the amyloid-β accumulation that is believed to be responsible for mitochondrial biogenesis disruption, and enhance mitochondrial respiratory capacity by increasing the expression of respiratory complexes I, III, and IV.^145^–^147^

As for future therapeutic interventions, a potential molecule that deserves additional study is mitochonic acid-5, a synthetic derivative of indole-3-acetic acid. Mitochonic acid-5 has been shown both *in vitro*, when added to human fibroblasts derived from mitochondrial diseases, and *in vivo*, when used in a murine model of IBM, to improve mitochondrial dynamics and decrease mitochondrial production of ROS.^55^,^148^

Another intervention that has been gaining momentum in the past couple of years is the transfer of healthy mitochondria, aiming to rescue or replace diseased mitochondria.^39^ The proposed mechanism is that the donor mitochondria could reduce the amount of mutated mitochondrial DNA in the recipient mitochondria.^149^ The technique is variable but mostly involves the delivery of autologous mitochondria from bone marrow mesenchymal cells, for instance, either intravenously or by direct injection into affected tissue.^150^,^151^ Since the second semester of 2021, a phase 1/2a clinical trial targeting DM/PM has been enrolling patients to define safety and tolerability of a single injection of allogeneic mitochondria (ClinicalTrials.gov Identifier: NCT04976140). Completion is expected by the middle of 2023.

## ANIMAL MODELS

In order to better define new treatment strategies, a proper understanding of disease mechanisms is essential. Of great assistance towards that aim is the use of animal models, particularly murine models, due to the relative simplicity of manipulating their genome.^152^ Gene manipulation permits a protein of interest to become constitutively active or, instead, suffer complete elimination either from the entire organism or from a pre-specified cell lineage.^153^ By doing that, one gains insight into the role of that protein on disease initiation and progression, and whether it is an attractive target for drug development.

An extensive overview of available animal models adopted for studying IIM is beyond the scope of this review, having been comprehensively described elsewhere.^154^ While there are well-characterized rabbit and dog models for IIM-like disease, most models use rodents, owing both to accessibility and similarity to the human genome.^155^

Regarding genetic engineering targeting specifically a mitochondria-related protein, it is worth mentioning the TDP-43 transgenic model.^156^ Either in its native or in its truncated isoform, TDP-43 has been found in greater quantities on muscle biopsy samples of IBM patients as compared to healthy controls.^157^ Aggregates of TDP-43 have been proposed to interfere with mitochondria bioenergetics, and, in the murine model in which this protein is overexpressed, machinery related to mitochondrial fission is increased, while that related to mitochondrial fusion is decreased,^158^ suggesting that imbalance of mitochondrial dynamics underlies the mechanism through which protein aggregates exert their pathogenic effect on muscle.

Similarly, accumulation of intramyofiber amyloid-β represents another interesting model for unraveling IBM pathophysiology.^159^ The amyloid-β fiber is the cleavage product of amyloid precursor protein, released as both 40 and 42 amino acid peptides, being present in up to 90% of vacuolated fibers, when human muscle biopsies are analyzed.^160^ More than a disease marker, the cytotoxic potential of amyloid-β has been demonstrated in neurodegenerative disorders.^161^

Based on findings from the MCK-β-amyloid precursor protein mouse model, the prototype for the accumulation of amyloid precursor protein proteolytic fragments in muscle tissue, it was shown that amyloid-β aggregates compromise the mitochondrial tricarboxylic acid cycle, forcing muscle cells to switch to anaerobic metabolism. Ultimately, this may lead to a reduction of cellular adenosine triphosphate levels, which could explain the loss of muscle strength observed.^162^

Finally, one model stands out when studying the relevance of mitochondria dysregulation for calcinosis, a common extra-muscular sign of DM. It has been suggested that mitochondria may be the cellular site in which calcium salts assemble first during the initiation of the dystrophic calcinosis process.^163^ Zhao et al. demonstrated that mitochondria become engorged and replete of calcific deposits after skeletal muscle is injured by cardiotoxin, both in a tumor necrosis factor-α receptor double-knockout mice model, and in an osteopontin-knockout mice model.^164^

## CONCLUSION

**Figure 2:**
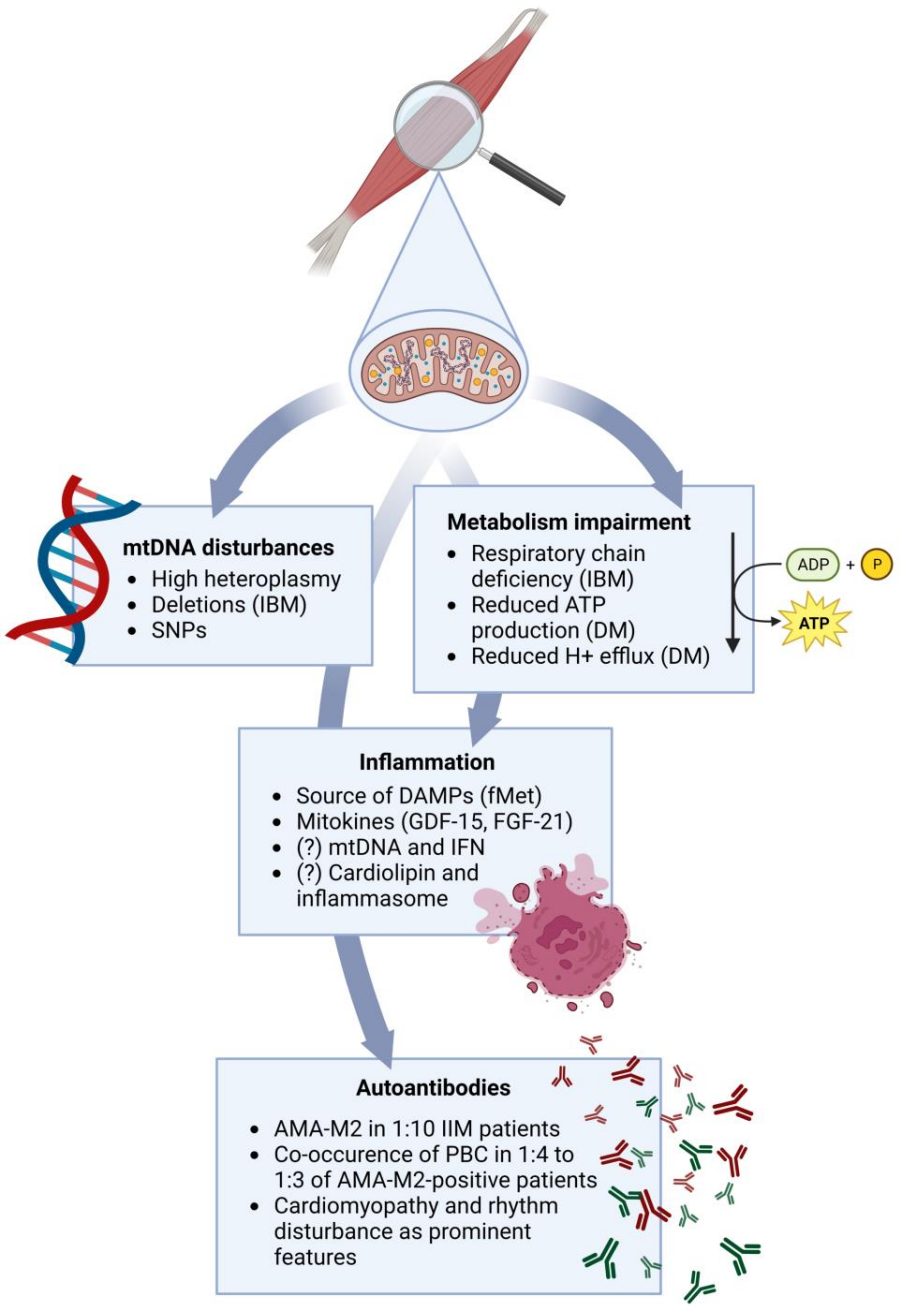
Main Proposed Mechanisms of How Mitochondria Influence the Pathophysiology of Idiopathic Inflammatory Myopathies. AMA-M2, anti-mitochondrial antibodies against the E2 subunit of the 2-oxo acid dehydrogenase complex; ATP, adenosine triphosphate; DAMPs, damage-associated molecular patterns; DM, dermatomyositis; FGF-21, fibroblast growth factor 21; fMet, N-formylated methionine peptides; GDF-15, growth differentiation factor-15; IBM, inclusion-body myositis; IFN, interferon; IIM, idiopathic inflammatory myopathy; mtDNA, mitochondrial DNA; PBC, primary biliary cholangitis; SNPs, single nucleotide polymorphisms. Created with Biorender.com.

As knowledge on the field has expanded and gained depth, the old traditional classification of IIM into the broad categories of “PM” and “DM” has been revised to include more diverse categories.^113^ By further understanding the link between MSA and disease phenotype, the all-encompassing terminology “DM” may actually drop in favor, as already happened to PM,^165^ since different MSA leads to particular disease manifestations.^166^ In that sense, AMA-positive IIM, which has started to be well characterized just in the last decade, may in the near future be set apart as a distinctive type of DM, as is already the case for anti-Mi-2, anti-NXP-2, anti-TIF-1γ, and anti-MDA-5 DM.^162^ As such, understanding of the role mitochondria play in disease pathogenesis—either as a source of antigenic load for AMA, or, one step back, as a crucial element in triggering innate immunity by ROS production and release of mitokines—should necessarily be on the research agenda of those interested in elucidating the disease mechanisms involved in IIM (Figure 2). Only by accruing information on the intricacies of mitochondrial dysfunction can we advance and foster new treatment modalities.

## Abbreviations

AMA(s): anti-mitochondrial antibody(ies)
ATP: adenosine triphosphate
COX: cytochrome c oxidase
DM: dermatomyositis
DNA: deoxriboynucleic acid
IBM: inclusion-body myositis
IIM: idiopathic inflammatory myopathies
MSA: myositis-specific antibodies
mtDNA: mitochondrial deoxriboynucleic acid
PM: polymyositis
RNA: ribonucleic acid
ROS: reactive oxygen species
rRNA: ribosomal RNA
tRNA: transfer RNA
TDP-43: transactivation response DNA-binding protein 43
